# Post-Artesunate Delayed Hemolysis: A Review of Current Evidence

**DOI:** 10.3390/tropicalmed8010049

**Published:** 2023-01-07

**Authors:** Sawettachai Jaita, Krit Madsalae, Sakarn Charoensakulchai, Borimas Hanboonkunapakarn, Kesinee Chotivanit, Anne E. McCarthy, Wasin Matsee

**Affiliations:** 1 Thai Travel Clinic, Hospital for Tropical Diseases, Faculty of Tropical Medicine, Mahidol University, Bangkok 10400, Thailand; 2 Chulabhorn Hospital, Chulabhorn Royal Academy, Bangkok 10210, Thailand; 3 Department of Parasitology, Phramongkutklao College of Medicine, Bangkok 10400, Thailand; 4 Department of Clinical Tropical Medicine, Faculty of Tropical Medicine, Mahidol University, Bangkok 10400, Thailand; 5 The Academy of Science, The Royal Society of Thailand, Dusit, Bangkok 10300, Thailand; 6 Department of Medicine, Faculty of Medicine, University of Ottawa, Ottawa, ON K1N 6N5, Canada

**Keywords:** malaria, post-artesunate delayed hemolysis, artesunate, hemolysis, anemia, treatment

## Abstract

Artesunate is the drug of choice for treating patients with severe malaria. Post-artesunate delayed hemolysis (PADH) is an uncommon adverse event from malaria treatment. Most patients with PADH are non-immune travelers. The pathophysiology of PADH is not fully understood, but the most likely mechanism is “pitting”, in which red blood cells carrying dead parasites killed by artesunate’s action are directed to the spleen for clearing the dead parasites. After the cleansing process, these red blood cells re-enter the circulation but with a smaller size and impaired integrity, resulting in a shortened lifespan of 7–21 days. Therefore, most patients with PADH usually present with clinical features of hemolytic anemia 7 days or later after the initiation of artesunate. To date, the benefits of artesunate treatment outweigh its adverse events, and no fatal cases have resulted from PADH. However, the hematological follow-up of patients with malaria treated with artesunate is recommended for clinicians to detect any delayed hemolytic event early and prevent potentially serious consequences.

## 1. Introduction

Malaria, which is a mosquito-borne protozoan infection, remains one of the most important global health problems among people living in the tropics. According to the World Malaria report in 2021, malaria cases have risen from 227 million in 2019 to 241 million in 2020. This increased figure is most likely due to medical service disruptions affected by the coronavirus disease 2019 pandemic [1].

Severe malaria is a life-threatening clinical spectrum associated with multi-organ dysfunction and is predominantly caused by *Plasmodium falciparum* infection [1]. However, a small proportion of complicated cases are due to other non-falciparum malaria infections [2,3,4]. This disease can become fatal if timely and accurate management is not provided.

The parenteral form of artemisinin derivatives, such as artesunate or artemether, was originally extracted from the Chinese medicinal plant *Artemisia annua* (Qinghaosu) [5]. These derivatives have been introduced as the treatment of choice for severe falciparum and non-falciparum malaria in adults, children, and pregnant women since 2006 [6].

Major randomized, clinical trials have shown that the efficacy of artesunate is superior to that of quinine, which is the previous first-line medication for severe malaria. Artesunate significantly decreased the mortality rate of adults with severe falciparum malaria in Asia to 35% (from 22% to 15%), and that of children in Africa to 22% (from 10.9% to 8.5%) compared with treatment with quinine [7,8]. Artesunate also showed a preferable safety profile in these studies. Although quinine is effective for the treatment of severe malaria, serious complications, such as hyperinsulinemic hypoglycemia, cardiotoxicity, and hypotension, frequently occur owing to its narrow therapeutic window. Therefore, quinine has been decreasingly prescribed, whereas artesunate has subsequently been the treatment of choice for complicated malaria over the past decade, especially in North America, Europe, and Southeast Asia [7,9,10,11].

After intravenous artesunate use was implemented worldwide, several incidences of delayed-onset hemolytic anemia were reported. Hemolysis usually occurs 7–30 days after the initiation of artemisinin-based therapy. This phenomenon is called post-artesunate or artemisinin-delayed hemolysis (PADH). This condition has been found in patients who live in endemic areas [12,13] and in non-immune travelers, most of whom had been diagnosed with severe falciparum malaria [14,15]. The objective of this review is to outline the clinical significance of PADH, with a focus on new hypotheses, plausible pathophysiology, and new strategies for predicting PADH and its proper management.

## 2. Epidemiology

Based on the finding of previous studies, the prevalence of PADH is considerably lower in patients living in endemic countries than in those living in non-endemic countries. A study reported that the incidence of PADH was doubled in European patients, compared with those of African origin [16]. Previous research in Africa showed only a 5% prevalence of PADH in children treated with intravenous artesunate [12]. On the other hand, studies performed on non-immune travelers demonstrated the prevalence of PADH to be as high as 50%, and more than half of the affected cases required blood transfusion [16,17]. Hyperparasitemia in severe malaria, with more than 5% of infected erythrocytes, is considered a major risk factor for PADH [6,14]. The occurrence of PADH is most commonly reported with the use of the intravenous form of artemisinin derivatives, but it has also been reported in those treated with oral artemisinin-based combination therapy, intramuscular injection, and the rectal form [18,19,20]. Despite the increasing number of PADH cases, with its severity ranging from mild to severe, no fatal outcome has been caused by PADH to date [21]. 

According to the meeting of the Medicines for Malaria Ventures in 2013, experts recommend that artesunate should be continued as the mainstay treatment of severe malaria. However, physicians need to recognize the condition of post-treatment hemolytic anemia or PADH because it can develop up to 1 month after the initiation of artesunate [18]. Little information is available on PADH. Therefore, the World Health Organization emphasizes the importance of pathophysiological and prospective clinical trials to identify the mechanisms, prognostic factors, frequency, magnitude of severity, and exact time course of PADH [18]. A substantial number of prospective studies have been conducted on PADH, with variable results [13,22]. 

## 3. Pathophysiology

**Figure 1:**
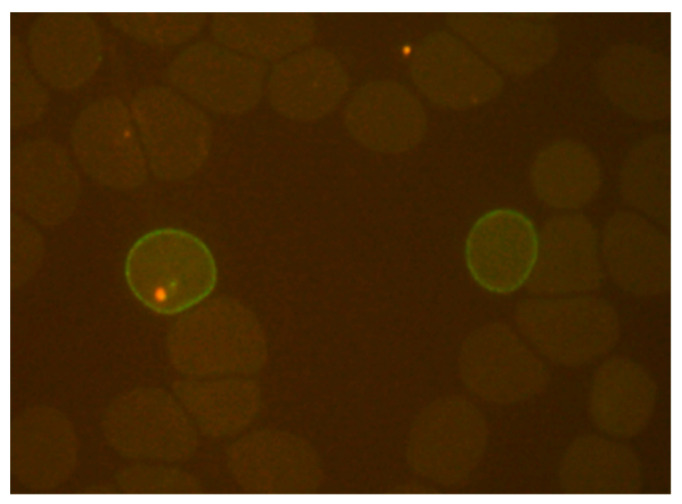
Immunofluorescence of peripheral blood film of *P. falciparum*-infected patients’ blood with post-artesunate delayed hemolysis. *P. falciparum*-infected red cells show positive staining with a monoclonal antibody to ring-infected erythrocyte surface antigen. The parasite was stained by acridine orange (**left**), and pitted red cells can be seen (**right**) (×1000 magnification).

The pathophysiological mechanisms of PADH have been extensively studied. However, the exact mechanism of PADH is currently inconclusive [23]. Currently, the process called “pitting” is thought to be the most possible pathophysiology of PADH [21,24], and the pitting mechanism is believed to be associated with the pharmacokinetics of the drug itself (Figure 1).

Artemisinin derivatives are categorized as an endoperoxide moiety. Once artemisinin derivatives are distributed within the patient’s circulation, this drug elicits the cleavage of an endoperoxide bridge, which subsequently contributes to the production of reactive oxygen species and free radicals [25]. These toxic byproducts directly harm the viability of intra-erythrocytic malarial parasites and contribute to the eradication of young-stage malarial parasites, also known as ring-form trophozoites. Artemisinin derivatives have robust and rapid erythrocytic schizonticidal activity against *Plasmodium* spp. [7,8,17,21,26,27]. Therefore, artemisinin derivatives mitigate the risk of erythrocyte sequestration and reduce the development of severe malaria [25]. After malarial parasites are killed, the erythrocytes that still contain the dead malarial parasites undergo the parasite removal process via splenic inter-endothelial slits. This phenomenon occurring inside the spleen is called pitting [25,26,28].

**Figure 2:**
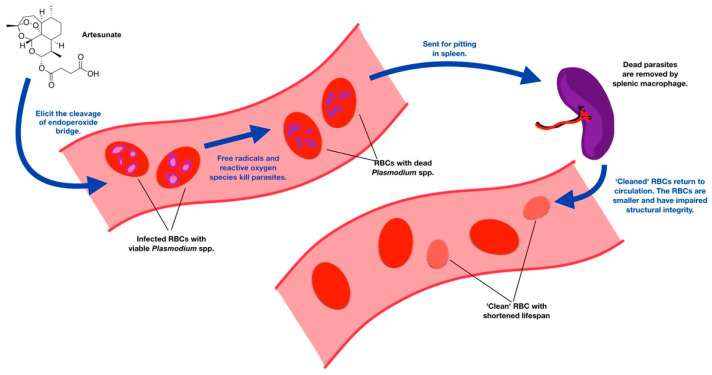
Schema of the pitting mechanism. Artesunate in circulation elicits the cleavage of an endoperoxide bridge, which produces free radicals and reactive oxygen species. These products kill trophozoites of *Plasmodium* spp. within RBCs. The RBCs that carry dead parasites are directed to the spleen to remove the dead parasites. After “cleaning” by splenic macrophages, RBCs are returned to circulation, but their lifespan is shortened to 7–21 days owing to an impaired cell structure. This phenomenon explains why PADH usually occurs 1 week or later after artesunate treatment in patients with malaria.

The mechanism of pitting aids in the removal of dead parasites without causing hemolysis [17,25,29]. The debris of dead organisms is later engulfed by splenic macrophages, whereas the intact “once-infected” red blood cells travel back to the host’s circulation. However, pitting causes the once-infected red blood cells to become smaller with impaired structural integrity [27]. As a result, the life span of these once-infected red blood cells becomes considerably shortened, to approximately 7–21 days [28]. This mechanism is more frequently found with a higher pitting rate in artesunate-treated patients than in those who are treated with quinine [28,29]. Therefore, PADH is more likely to occur in artesunate-treated patients with a higher parasitemia level because there is a larger number of short-lived pitted red blood cells [26]. A cohort study conducted in western Africa confirmed this likelihood by showing that those with higher geometric mean parasite densities (GMPD) resulted in a higher incidence of hemolytic anemia on day 14 than those with lower GMPD (306,968/µL vs. 92,642/µL) [14]. However, PADH has also been reported in individuals with a low parasite count. The pitting mechanism is shown in Figure 2.

PADH is not only caused by the destruction of once-infected pitted cells but also by the destruction of uninfected red blood cells. In some cases, hemoglobin concentrations unexpectedly rapidly decrease. There may also be artesunate-suppressed hematopoietic precursor cells in bone marrow, causing the impairment of terminal erythroid differentiation via a direct effect on erythroblasts and indirectly via macrophages, as well as reticulocyte maturation. Several in vitro and in vivo studies in both human cells and animals suggested that artemisinin and derivatives have the dose-dependent inhibition of the proliferation of proerythroblasts and basophilic erythroblasts and erythroid differentiation effects. These effects consequently decrease erythrocyte and reticulocyte counts, a sensitive measure of erythropoiesis inhibition. Reticulocyte depletion is reversible and can return to a normal or higher level after the therapy. Therefore, the inhibition of erythropoiesis may contribute to PADH in patients receiving a high dose of artesunate [30,31,32,33].

Another proposed mechanism of PADH is drug-induced immune hemolytic anemia. That is because the pitting mechanism alone cannot explain the occurrence of delayed hemolysis in splenectomized patients. Furthermore, there have been increasing cases of PADH with positive direct antiglobulin test (DAT), which were successfully treated with corticosteroid administration [23,34,35]. This positive result of DAT suggests the possibility of autoimmune hemolytic anemia (AIHA) [36]. The etiology of hemolytic anemia can be either idiopathic or secondary to certain causes including medication. Scientists suggested the plausible pathophysiology explained by drug–anti-drug immune complex formation on the red blood cell surface; thus, the production of autoantibodies causes positive DATs and immune hemolytic anemia [20,37].

However, whether the positive DAT is an exclusive outcome of artesunate remains debatable. Apart from directly invading erythrocytes, malaria parasites inherently induce autoantibodies against “phosphatidylserine”, which is a surface antigen coated on uninfected erythrocytes of malaria-infected patients causing anemia during the active phase of malaria [38]. These autoantibodies expressed once infected may persist in the circulation, which may be detected later when PADH is suspected. For this reason, a prospective observational study to evaluate immune-mediated hemolytic anemia as a result of artemisinin treatment is paramount [39]. 

Bone marrow suppression has been hypothesized as one of the plausible etiologies of PADH as well [30]. An In vivo study found a decrease in reticulocyte counts after administering intravenous artesunate 4 mg/kg in healthy adults [40]. This is perhaps due to a temporary arrest of the basophilic erythroblast stage of erythrocytes resulting in low reticulocyte count, which leads to the early onset of anemia during active malaria. Once the anemia reaches its nadir phase, the bone marrow would be activated causing a rebound of erythropoiesis, which subsequently brings about a rising level of reticulocytes at the stage of PADH [41]. Anemia due to artemisinin was also demonstrated in animal models. A study conducted on rats showed mild anemia after giving a high dose of oral artemisinin (50–100 mg/kg/day) at 2-week and 4-week follow-ups [42,43]. An In vitro study using a leukemia cell line also confirmed that artemisinin derivatives inhibit erythroid differentiation [33].

## 4. Clinical Features and Laboratory Results

Most of the patients with PADH experience severe malaria with hyperparasitemia and are treated with artemisinin derivatives. The characteristics of patients with late-onset hemolysis are not different from those with other causes of hemolytic anemia, which include anemia, jaundice, and dark urine [18,44,45]. There needs to be an awareness of the overlapping clinical features between PADH and hemolytic anemia caused by malaria infection, such as blackwater fever and severe hemolysis. However, patients without clinical symptoms, but with a hemolytic anemia profile, have also been reported occasionally during malaria post-treatment follow-up visits [27,44,46]. PADH seems to be dose-independent [46]. There was no evidence that supports the relationship between delayed parasite clearance and the development of PADH despite longer treatment duration.

Currently, no official standard diagnostic criteria for PADH are available [17]. A patient should be suspected as having PADH if a new onset of hemolytic anemia without parasitemia develops more than 7 days after the initiation of artemisinin derivatives. This typical history is commonly known as the delayed type of PADH. Another pattern is called persistent PADH, in which a decline in the hemoglobin concentration is found at approximately day 7 after the beginning of artemisinin medication. The anemia remains constant and persists beyond day 14 [20,24,25]. Most importantly, other potential causes of hemolytic anemia must be excluded (Table 1).

**Table 1:** Differential diagnosis of PADH [47,48].

Acquired	Hereditary
Mechanical (1) Microangiopathic (disseminated intravascular coagulation, thrombotic thrombocytopenic purpura, and vasculitis) (2) Parasites and microorganisms (e.g., malaria, black water fever, babesiosis, bartonellosis, *Clostridium perfringens*, *Rickettsia, Hemophilus influenzae*, and human immunodeficiency virus) (3) Drug-induced (drug-induced thrombotic microangiopathy, drug-induced immune hemolytic anemia, and oxidative hemolysis) b. Antibody-mediated (1) Warm and cold autoimmune hemolytic anemia (2) Transfusion reactions (immediate and delayed)	Hemoglobinopathies (sickle cell disease, thalassemia, and hemoglobin defects) Red cell membrane disorders (e.g., hereditary spherocytosis, paroxysmal nocturnal hemoglobinuria, and hereditary elliptocytosis) Red cell enzyme defects (glucose-6-phosphate dehydrogenase or pyruvate kinase deficiencies)

The laboratory results need to be consistent with the profile of hemolytic anemia (Table 2). Hemolytic anemia is defined as a 10% decrease in hemoglobin concentrations, haptoglobin concentrations < 0.1 g/L, and an increase in the lactate dehydrogenase concentration to 390 IU/L or a rise in lactate dehydrogenase concentration of at least 10% after starting artesunate for more than 7 days [26]. Apart from the hemolytic anemia profile, few studies have reported dose-dependent neutropenia in patients treated with artesunate [46,49]. A summary of case reports of PADH is shown in Table 3.

**Table 2:** Initial laboratory results found in a patient with PADH. (Adapted with permission from ref. [47], Copyright 2018).

Test	Finding	Cause
Haptoglobin concentrations	Low or absent	Binds free hemoglobin (Hb)
Lactate dehydrogenase concentration	High	Released from lysis of RBCs
Reticulocyte count	High	Marrow response to anemia
Unconjugated bilirubin	High	Increased Hb breakdown
Urinalysis	Urobilinogen, positive for blood	Free Hb and its metabolites

**Table 3:** Characteristics of PADH patients in case reports.

	Jarvis et al. [50]	Raffray et al. [23]	Paczkowski et al. [51]	Plewes et al. [52]	Boillat et al. [27]	Salehi et al. [53]	Conlon et al. [20]	Patel et al. [54]	Matsee et al. [45]	Martino et al. [46]
Participant(s)	1 adult returned traveler	1 adult returned traveler	2 adults returned traveler	1 adult endemic area	4 adults returned traveler	1 adult returned traveler	1 adult returned traveler	1 child endemic area	1 adult returned traveler	1 child returned traveler
Year	2013	2014	2014	2015	2015	2019	2020	2020	2021	2022
Day of hemolysis detection	8	8	9–11	14	8–28	12	8	11	15	7
Severity	Severe without hyperparasitemia	Severe without hyperparasitemia	Severe with hyperparasitemia	Severe with hyperparasitemia	Severe with and without hyperparasitemia	Severe with hyperparasitemia	Severe with hyperparasitemia	Severe without hyperparasitemia	Not severe	Severe with hyperparasitemia
Sign/Symptom	Fever, dyspnea, ankle swelling, anemia, jaundice, tachycardia, hepatosplenomegaly, dark urine	Fever, fatigue	Weakness, fatigue, SOB, leg edema	Fever, rigors, headache, N/V, abdominal pain, jaundice	Fever, fatigue, jaundice, SOB	Fever, jaundice, headache, dark urine	Fever, N/V	Fever, weakness, loss of appetite, headache, anemia, jaundice, tachycar-dia, dark urine	Malaise, fatigue	Asymptomatic
Laboratory findings	Hb 4.1 g/dL, haptoglobin < 0.1 g/L, Normal LFT, High LDH	Hb 4.6 g/dL, high LDH, absent haptoglobin	Hb 5.7–6.8 g/dL, high LDH, TB, and reticulocyte count	Hb 4.9 g/dL, high LDH, decreased haptoglobin, normal G6PD	Hb 5.6–12.4 g/dL, high LDH, low haptoglobin, high reticulocyte count	Hb 4.7 g/dL, high TB, LDH, 3994 U/L negative Coombs test, normal reticulocyte count	Hb 10.2 g/dL, low haptoglobin < 0.1 g/L, high LDH 1759 U/L, rising TB	Hb 10 g/dL, hemolytic blood picture, high reticulocyte count, high LDH, parasitemia	Hb 9.1 g/dL LDH 1706 U/L, low haptoglobin (<0.024 mg/mL), high reticulocyte count	Hb 7.7 g/dL, high reticulocyte count, haptoglobin < 0.08 g/dL
Laboratory findings at the follow-up	No data	*Follow-up Day 52* Hb > 12 g/dL Haptoglobin and LDH were within normal limits	*Follow-up Day 19* Hb 9.8 g/dL in one case No data of another case	No data	*Follow-up Day 28–35* Hb 10.5–14 g/dL Low LDH	No data	No data	*Follow-up after 1 month* Hb 134 g/L Other hematological parameters returned to normal range	*Follow-up Day 29* Hb 10.2 g/dL *Follow-up* *2 months* Hb 13.3 g/dL LDH 355 U/L	*Follow-up* *day 21* Hb 9.2 mg/dL
Blood transfusion	Yes	No	Yes	Yes	Yes	Yes	Yes	Yes	No	N/A

SOB = shortness of breath, N/V = nausea and vomiting, Hb = hemoglobin, LFT = liver function test, LDH = lactate dehydrogenase, TB = total bilirubin.

The positivity of direct antiglobulin tests has been reported in several PADH cases, suggesting drug-induced immune hemolytic anemia. When hemolysis is highly suspected, a DAT test should be performed to distinguish immune-related hemolytic anemia from other non-immune causes. A published case series reported that almost half of individuals with PADH had a positive DAT test [39]. DAT results were reported in detail in 13 of the 17 (76.5%) positive participants. Five (38.5%) of them showed an immunoglobulin G pattern, six (46.1%) presented with positivity for complement component C3d, and the remaining two (15.4%) were characterized by a mixed condition. However, this study concluded that late-onset hemolytic anemia was not associated with DAT positivity.

## 5. Role of the *P. falciparum*-Histidine-Rich Protein 2 Rapid Diagnostic Test for Predicting Subsequent PADH

Histidine-rich protein 2 (HRP2), which is a component of a histidine- and alanine-rich protein specifically produced by *P. falciparum*, is related to parasite proliferation and growth. HRP2 protein in once-infected erythrocytes remains positive for several weeks after parasite clearance. 

A recent study adapted the widely used *P. falciparum* histidine-rich protein 2 (pf-HRP2) rapid diagnostic test to alternatively measure pf-HRP2 protein concentrations as a predictive marker for PADH in artesunate-treated patients. A previous study showed that pf-HRP2 protein concentrations in the whole blood of artesunate-treated patients were higher than those in patients treated with quinine [31]. Using a 1:500 dilution of the whole blood collected from artesunate-treated patients after recent parasite clearance, the positivity in an HRP2-based rapid diagnostic dipstick test showed promising efficacy in detecting PADH, with a sensitivity of 89% and a specificity of 73%. The results of this study may be a milestone in the development of a reliable diagnostic tool for the early detection of PADH in the near future. 

## 6. Implementation

Although the potential risk of post-treatment hemolysis after the use of artesunate and other artemisinin derivatives is clearly evident in a substantial number of patients, particularly in non-immune patients, this concern should not be a reason to withhold this medication [18]. After discharge, weekly follow-up of artesunate-treated patients is recommended to clinicians for the monitoring and early detection of delayed hemolytic events. In each visit, a physician should search for signs and symptoms of hemolytic anemia, as well as perform a laboratory investigation for the early detection of hemolytic anemia, such as a complete blood count, serum haptoglobin concentrations, or lactate dehydrogenase concentrations [26]. Although fatality from PADH has not been reported, this condition may contribute to life-threatening anemia in some cases. In countries where safe blood products are not widely available, this event can cause a great burden on their healthcare system [21]. Therefore, vigilance for the detection of PADH, especially for non-immune patients with a high parasitemia level, is crucial. 

## 7. Conclusions and Future Directions

This review highlights the requirement for the ongoing vigilance of post-treatment hemolysis in patients treated with artemisinin derivatives, regardless of the height of their parasite count [13]. Clinicians need to appreciate that PADH might emerge from more than one process, and other potential mechanisms and several contributing factors should also be taken into consideration. Moreover, a close follow-up of patients after malaria treatment is important to diagnose and differentiate PADH from other hemolytic anemia conditions. A complete blood count should be performed, and malaria should be monitored with microscopy at 7, 14, 21, and 28 days after artemisinin administration to determine if PADH is present and for the recrudescence of *P. falciparum* malaria. More information on the pathophysiological mechanisms of PADH would improve the management of these patients.

## Data Availability

Not Applicable.

## References

[1] World Health Organization (2021). World Malaria Report.

[2] Kotepui M., Kotepui K.U., Milanez G.D., Masangkay F.R. (2020). Severity and mortality of severe Plasmodium ovale infection: A systematic review and meta-analysis. PLoS ONE.

[3] Rajahram G.S., Barber B.E., William T., Menon J., Anstey N.M., Yeo T.W. (2012). Deaths due to Plasmodium knowlesi malaria in Sabah, Malaysia: Association with reporting as Plasmodium malariae and delayed parenteral artesunate. Malar. J..

[4] Rahimi B.A., Thakkinstian A., White N.J., Sirivichayakul C., Dondorp A.M., Chokejindachai W. (2014). Severe vivax malaria: A systematic review and meta-analysis of clinical studies since 1900. Malar. J..

[5] Brown G.D. (2010). The Biosynthesis of Artemisinin (Qinghaosu) and the Phytochemistry of *Artemisia annua* L. (Qinghao). Molecules.

[6] World Health Organization (2010). Guidelines for Malaria Treatment. https://apps.who.int/medicinedocs/documents/s19105en/s19105en.pdf.

[7] Dondorp A., Nosten F., Stepniewska K., Day N., White N. (2005). South East Asian Quinine Artesunate Malaria Trial (SEAQUAMAT) group Artesunate versus quinine for treatment of severe falciparum malaria: A randomised trial. Lancet.

[8] Dondorp A.M., Fanello C.I., Hendriksen I.C., Gomes E., Seni A., Chhaganlal K.D., Bojang K., Olaosebikan R., Anunobi N., Maitland K. (2010). Artesunate versus quinine in the treatment of severe falciparum malaria in African children (AQUAMAT): An open-label, randomised trial. Lancet.

[9] Kurth F., Develoux M., Mechain M., Malvy D., Clerinx J., Antinori S., Gjørup I.E., Gascon J., Mørch K., Nicastri E. (2017). Severe malaria in Europe: An 8-year multi-centre observational study. Malar. J..

[10] (2020). Committee to Advise on Tropical Medicine and Travel. Canadian Recommendations for the Prevention and Treatment of Malaria. https://www.canada.ca/en/public-health/services/catmat/canadian-recommendations-prevention-treatment-malaria.html.

[11] Centers for Disease Control and Prevention (2017). CDC Yellow Book 2020: Health Information for International Travel.

[12] Scheu K., Adegnika A.A., Addo M.M., Ansong D., Cramer J.P., Fürst S., Kremsner P.G., Kurth F., Jacobs T., May J. (2019). Determinants of post-malarial anemia in African children treated with parenteral artesunat. Nat. Rev..

[13] Savargaonkar D., Das M.K., Verma A., Mitra J.K., Yadav C.P., Srivastava B., Anvikar A.R., Valecha N. (2020). Delayed haemolysis after treatment with intravenous artesunate in patients with severe malaria in India. Malar. J..

[14] Rolling T., Agbenyega T., Issifou S., Adegnika A.A., Sylverken J., Spahlinger D., Ansong D., Löhr S.J., Burchard G.D., May J. (2014). Delayed hemolysis after treatment with parenteral artesunate in African children with severe malariaea double-center prospective study. J. Infect. Dis..

[15] Jauréguiberry S., Thellier M., Ndour P.A., Ader F., Roussel C., Sonneville R., Mayaux J., Matheron S., Angoulvant A., Wyplosz B. (2015). Delayed-Onset Hemolytic Anemia in Patients with Travel-Associated Severe Malaria Treated with Artesunate, France, 2011–2013. Emerg. Infect. Dis..

[16] Roussel C., Ndour P.A., Kendjo E., Larréché S., Taieb A., Henry B., Lebrun-Vignes B., Chambrion C., Argy N., Houzé S. (2021). Intravenous Artesunate for the Treatment of Severe Imported Malaria: Implementation, Efficacy, and Safety in 1391 Patients. Clin. Infect. Dis..

[17] Roussel M.C., Caumes E., Thellier M., Ndour P.A., Buffet P., Jauréguiberry S. (2017). Artesunate to treat severe malaria in travellers: Review of efficacy and safety and practical implications. J. Travel Med..

[18] World Health Organization (2013). WHO Information Note on Delayed Haemolytic Anaemia Following Treatment with Artesunate. https://www.who.int/malaria/publications/atoz/who_note_delayed_haemolytic_anaemia_oct13.pdf?ua=1.

[19] Rolling T., Wichmann D., Schmiedel S., Burchard G.D., Kluge S., Cramer J.P. (2013). Artesunate versus quinine in the treatment of severe imported malaria: Comparative analysis of adverse events focussing on delayed haemolysis. Malar. J..

[20] Conlon C.C., Stein A., Colombo R.E., Schofield C. (2020). Post-artemisinin delayed hemolysis after oral therapy for *P. falciparum* infection. Idcases.

[21] Rehman K., Lötsch F., Kremsner P.G., Ramharter M. (2014). Haemolysis associated with the treatment of malaria with artemisinin derivatives: A systematic review of current evidence. Int. J. Infect. Dis..

[22] Kurth F., Lingscheid T., Steiner F., Stegemann M.S., Bélard S., Menner N., Pongratz P., Kim J., von Bernuth H., Mayer B. (2016). Hemolysis after Oral Artemisinin Combination Therapy for Uncomplicated *Plasmodium falciparum* Malaria. Emerg. Infect. Dis..

[23] Raffray L., Receveur M., Beguet M., Lauroua P., Pistone T., Malvy D. (2014). Severe delayed autoimmune haemolytic anaemia following artesunate administration in severe malaria: A case report. Malar. J..

[24] Chotivanich K., Udomsangpetch R., Dondorp A., Williams T., Angus B., Simpson J.A., Pukrittayakamee S., Looareesuwan S., Newbold C.I., White N.J. (2000). The Mechanisms of Parasite Clearance after Antimalarial Treatment of *Plasmodium falciparum* Malaria. J. Infect. Dis..

[25] Rolling T., Agbenyega T., Krishna S., Kremsner P., Cramer J. (2015). Delayed haemolysis after artesunate treatment of severe malaria e Review of the literature and perspective. Travel Med. Infect Dis..

[26] Arguin P.M. (2014). Case definition: Postartemisinin delayed hemolysis. Blood.

[27] Boillat O., Spechbach H., Chalandon Y., Eperon G. (2015). Post-artesunate delayed haemolysis–report of four cases and review of the literature. Swiss Med. Wkly..

[28] Jauréguiberry S., Ndour P.A., Roussel C., Ader F., Safeukui I., Nguyen M., Biligui S., Ciceron L., Mouri O., Kendjo E. (2014). Postartesunate delayed hemolysis is a predictable event related to the lifesaving effect of artemisinins. Blood.

[29] Ndour P.A., Larréché S., Mouri O., Argy N., Gay F., Roussel C., Jauréguiberry S., Perillaud C., Langui D., Biligui S. (2017). Measuring the *Plasmodium falciparum* HRP2 protein in blood from artesunate-treated malaria patients predicts post-artesunate delayed hemolysis. Sci. Transl. Med..

[30] Medicines for Malaria Ventures (2013). Experts Group Meeting on Delayed Haemolytic Anaemia Following Treatment with Injectable Artesunate. https://www.mmv.org/sites/default/files/uploads/docs/events/2013/InjectableArtesunateExpertGroupMeeting.pdf.

[31] Efferth T., Kaina B. (2010). Toxicity of the antimalarial artemisinin and its dervatives. Crit. Rev. Toxicol..

[32] Finaurini S., Ronzoni L., Colancecco A., Cattaneo A., Cappellini M.D., Ward S.A., Taramelli D. (2010). Selective toxicity of dihydroartemisinin on human CD34+ erythroid cell differentiation. Toxicology.

[33] Finaurini S., Basilico N., Corbett Y., D’Alessandro S., Parapini S., Olliaro P., Haynes R.K., Taramelli D. (2012). Dihydroartemisinin inhibits the human erythroid cell differentiation by altering the cell cycle. Toxicology.

[34] Camprubí D., Pereira A., Rodriguez-Valero N., Almuedo A., Varo R., Casals-Pascual C., Bassat Q., Malvy D., Muñoz J. (2019). Positive direct antiglobulin test in post-artesunate delayed haemolysis: More than a coincidence?. Malar. J..

[35] Lebrun D., Floch T., Brunet A., Julien G., Romaru J., N’Guyen Y., Cousson J., Giltat A., Toubas D., Bani-Sadr F. (2017). Severe post-artesunate delayed onset anaemia responding to corticotherapy: A case report. J. Travel Med..

[36] Parker V., Tormey C.A. (2017). The Direct Antiglobulin Test: Indications, Interpretation, and Pitfalls. Arch. Pathol. Lab. Med..

[37] Garratty G. (2010). Immune hemolytic anemia associated with drug therapy. Blood Rev..

[38] Barber B.E., Grigg M.J., Piera K., Amante F.H., William T., Boyle M.J., Minigo G., Dondorp A.M., McCarthy J., Anstey N.M. (2019). Antiphosphatidylserine Immunoglobulin M and Immunoglobulin G Antibodies Are Higher in Vivax than Falciparum Malaria, and Associated with Early Anemia in Both Species. J. Infect. Dis..

[39] Ascoli Bartoli T., Lepore L., D’Abramo A., Adamo G., Corpolongo A., Scorzolini L., Giancola M.L., Bevilacqua N., Palazzolo C., Mariano A. (2021). Systematic analysis of direct antiglobulin test results in post-artesunate delayed haemolysis. Malar. J..

[40] Weina P.J., Haeberle A.S., Milhous W.K. (2006). Intravenous Artesunate: The New Generation of Lifesaving Treatment for Severe Malaria in the Warfighter.

[41] Clark R.L. (2014). Hypothesized cause of delayed hemolysis associated with intravenous artesunate. Med. Hypotheses.

[42] Knights P. (2002). Artesunate: 14 Days Preliminary Oral (Gavage Administration) Toxicity Study in the Rat. Covance Report.

[43] Knights P. (2002). Artesunate: 4 Weeks Oral (Gavage Administration) Toxicity Study in the Rat. Covance Report.

[44] Gómez-Junyent J., Ruiz-Panales P., Calvo-Cano A., Gascón J., Muñoz J. (2017). Delayed haemolysis after artesunate therapy in a cohort of patients with severe imported malaria due to Plasmodium falciparum. Enferm. Infecc. Microbiol. Clin..

[45] Matsee W., Hiranrusme T., Pisutsan P., Hanboonkunupakarn B., Chotivanich K. (2021). Returned traveller presenting with anaemia: Clinical challenge of post-artesunate delayed haemolysis. J. Travel Med..

[46] Martino M., Liberati C., Bua B., Barbieri E., Costenaro P., Di Chiara C., Giaquinto C., De Canale E., Rampon O., Donà D. (2022). Treatment for Severe Malaria: Post-Artesunate Delayed Haemolysis and Neutropenia. Healthcare.

[47] Phillips J., Henderson A.C. (2018). Hemolytic Anemia: Evaluation and Differential Diagnosis. Am. Fam. Physician.

[48] Packman C. (2016). William Hematology.

[49] Bethell D., Se Y., Lon C., Khemawoot P., Darapiseth S., Sriwichai S., Kuntawungin W., Surasri S., Sarim S., Tyner S. (2010). Dose?Dependent Risk of Neutropenia after 7? Day Courses of Artesunate Monotherapy in Cambodian Patients with Acute Plasmodium falciparum Malaria. Clin. Infect. Dis..

[50] Jarvis J.N., Coltart C.E., Pule M., Chiodini P., Doherty T. (2013). Artemisinin therapy and severe delayed haemolysis. Lancet.

[51] Paczkowski M., Landman K., Arguin P. (2014). Update on cases of delayed hemolysis after parenteral artesunate therapy for malaria—United States, 2008 and 2013. MMWR Morb. Mortal Wkly. Rep..

[52] Plewes K., Haider S., Kingston H.W.F., Yeo T.W., Ghose A., Hossain A., Dondorp A.M., Turner G.D.H., Anstey N.M. (2015). Severe falciparum malaria treated with artesunate complicated by delayed onset haemolysis and acute kidney injury. Malar. J..

[53] Salehi M., Masoumi-Asl H., Assarian M., Khoshnam-Rad N., Haghi A.M., Nikbakht M., Khalili H. (2019). Delayed Hemolytic Anemia after Treatment with Artesunate: Case Report and Literature Review. Curr. Drug Saf..

[54] Patel N., Thomson J., Ferre L.R. (2020). Delayed haemolysis following artesunate in a child with profound anaemia and Coca-Cola-coloured urine. BMJ Case Rep..

